# Five patients with disorders of calcium metabolism presented with *GCM2* gene variants

**DOI:** 10.1038/s41598-021-82661-y

**Published:** 2021-02-03

**Authors:** Alejandro García-Castaño, Leire Madariaga, Sara Gómez-Conde, Carmen Lourdes Rey Cordo, María López-Iglesias, Yolanda Garcia-Fernández, Alicia Martín, Pedro González, Ignacio Goicolea, Gustavo Pérez de Nanclares, Ana Belén De la Hoz, Aníbal Aguayo, Idoia Martínez de LaPiscina, Rosa Martínez, Laura Saso, Inés Urrutia, Olaia Velasco, Luis Castaño, Sonia Gaztambide

**Affiliations:** 1 Biocruces Bizkaia Health Research Institute, CIBERDEM, CIBERER, Plaza de Cruces, Barakaldo, 48903 Bizkaia, Spain; 2 Biocruces Bizkaia Health Research Institute, CIBERDEM, CIBERER, Pediatric Nephrology Department, Hospital Universitario Cruces, University of the Basque Country (UPV/EHU), Bizkaia, Spain; 3 Biocruces Bizkaia Health Research Institute, Bizkaia, Spain; 4 Pediatric Endocrinology Department, Hospital Álvaro Cunqueiro, EOXI, Vigo, Spain; 5 Endocrinology Department, Hospital General La Mancha Centro, Ciudad Real, Spain; 6 Endocrinology and Nutrition Department, OSI Barrualde-Galdakao, Bizkaia, Spain; 7 Biocruces Bizkaia Health Research Institute, Endocrinology and Nutrition Department, Hospital Universitario Cruces, Bizkaia, Spain; 8 Biocruces Bizkaia Health Research Institute, CIBERDEM, CIBERER, Hospital Universitario Cruces, Bizkaia, Spain; 9 Biocruces Bizkaia Health Research Institute, CIBERER, Bizkaia, Spain; 10 Biocruces Bizkaia Health Research Institute, CIBERDEM, CIBERER, Endocrinology and Nutrition Department, Hospital Universitario Cruces, University of the Basque Country (UPV/EHU), Bizkaia, Spain

**Keywords:** Genetics, Endocrinology

## Abstract

The *GCM2* gene encodes a transcription factor predominantly expressed in parathyroid cells that is known to be critical for development, proliferation and maintenance of the parathyroid cells. A cohort of 127 Spanish patients with a disorder of calcium metabolism were screened for mutations by Next-Generation Sequencing (NGS). A targeted panel for disorders of calcium and phosphorus metabolism was designed to include 65 genes associated with these disorders. We observed two variants of uncertain significance (p.(Ser487Phe) and p.Asn315Asp), one likely pathogenic (p.Val382Met) and one benign variant (p.Ala393_Gln395dup) in the *GCM2* gene in the heterozygous state in five families (two index cases had hypocalcemia and hypoparathyroidism, respectively, and three index cases had primary hyperparathyroidism). Our study shows the utility of NGS in unravelling the genetic origin of some disorders of the calcium and phosphorus metabolism, and confirms the *GCM2* gene as an important element for the maintenance of calcium homeostasis. Importantly, a novel variant in the *GCM2* gene (p.(Ser487Phe)) has been found in a patient with hypocalcemia.

## Introduction

Calcium (Ca^2+^) is required for many physiological functions (muscle contraction, nerve conduction, hormone release, mineralization of bone, and blood coagulation). Ca^2+^ metabolism maintains a dynamic balance between intestinal absorption, exchange with the bone, and renal excretion. Parathyroid hormone (PTH), vitamin D and calcitonin regulate this balance by acting on their targets: intestine, bone and renal tubule. PTH is secreted by the parathyroid glands. The *GCM2* gene (glial cells missing transcription factor 2, MIM *603716) encodes a transcription factor predominantly expressed in parathyroid cells^1,2^. This transcription factor is known to be critical for development of the parathyroid cells and plays a critical role in adult parathyroid cell proliferation and maintenance^3,4^. Thus, it has been demonstrated that a *GCM2*-deficient mouse lacked parathyroid glands^5^.

The *GCM2* gene was mapped to chromosome 6p24.2^2^ and encodes the chorion-specific transcription factor GCMb. The full-length protein (506 amino acids) contains a N-terminal Zn-containing DNA binding domain (amino acids from 19 to 174), a nuclear localization sequence (amino acids from 176 to 191), a C-terminal conserved inhibitory domain (CCID, amino acids from 379 to 395), and two transcriptional activation domains in the C-terminal region: TAD1 (amino acids from 175 to 263) and TAD2 (amino acids from 428 to 506)^2,6–8^.

Loss-of-function mutations in the *GCM2* gene have recently been described in humans as a rare cause of autosomal dominant or recessive familial isolated type 2 hypoparathyroidism (MIM #618883)^6^. Patients usually develop seizures due to the hypocalcemia, in early life, with hyperphosphatemia, low to undetectable serum PTH levels and normal levels of 25-hydroxyvitamin D and 1,25-dihydroxyvitamin D. On the other hand, gain-of-function mutations in the *GCM2* gene cause autosomal dominant hyperparathyroidism type 4 (MIM #617343)^9^, a disorder characterized by hypercalcemia and elevated or inappropriate PTH secretion by parathyroid glands.

In the present study, we report a novel variant of uncertain significance in the *GCM2* gene in a family from Spain with severe hypocalcemia. Moreover, we report three previously described *GCM2* gene variants, one likely pathogenic, one of uncertain significance and one benign, in four families from Spain presenting with different disorders of calcium metabolism.

## Materials and methods

### Ethics statement

The study was approved by the Ethics Committee for Clinical Research of Euskadi (CEIC-E). Patients and their participating relatives provided written informed consent for the genetic study. The research was carried out in accordance with the Declaration of Helsinki on human experimentation of the World Medical Association.

### Patients

A total of 65 genes whose mutations are a recognized cause of calcium and phosphorus metabolism disorders were tested by a Next-Generation Sequencing (NGS) panel in a cohort of 127 Spanish patients (50 had hypocalciuric hypercalcemia, 44 were diagnosed of primary hyperparathyroidism, 13 presented with hypocalcemia and/or hypoparathyroidism, 12 were diagnosed of pseudohypoparathyroidism and 8 had rickets). Clinical diagnoses were made by adult and pediatric endocrinologists. In all cases, the molecular analysis was done in the Molecular Genetic Laboratory at Biocruces Bizkaia Health Research Institute, Barakaldo, Spain.

**Figure 1:**
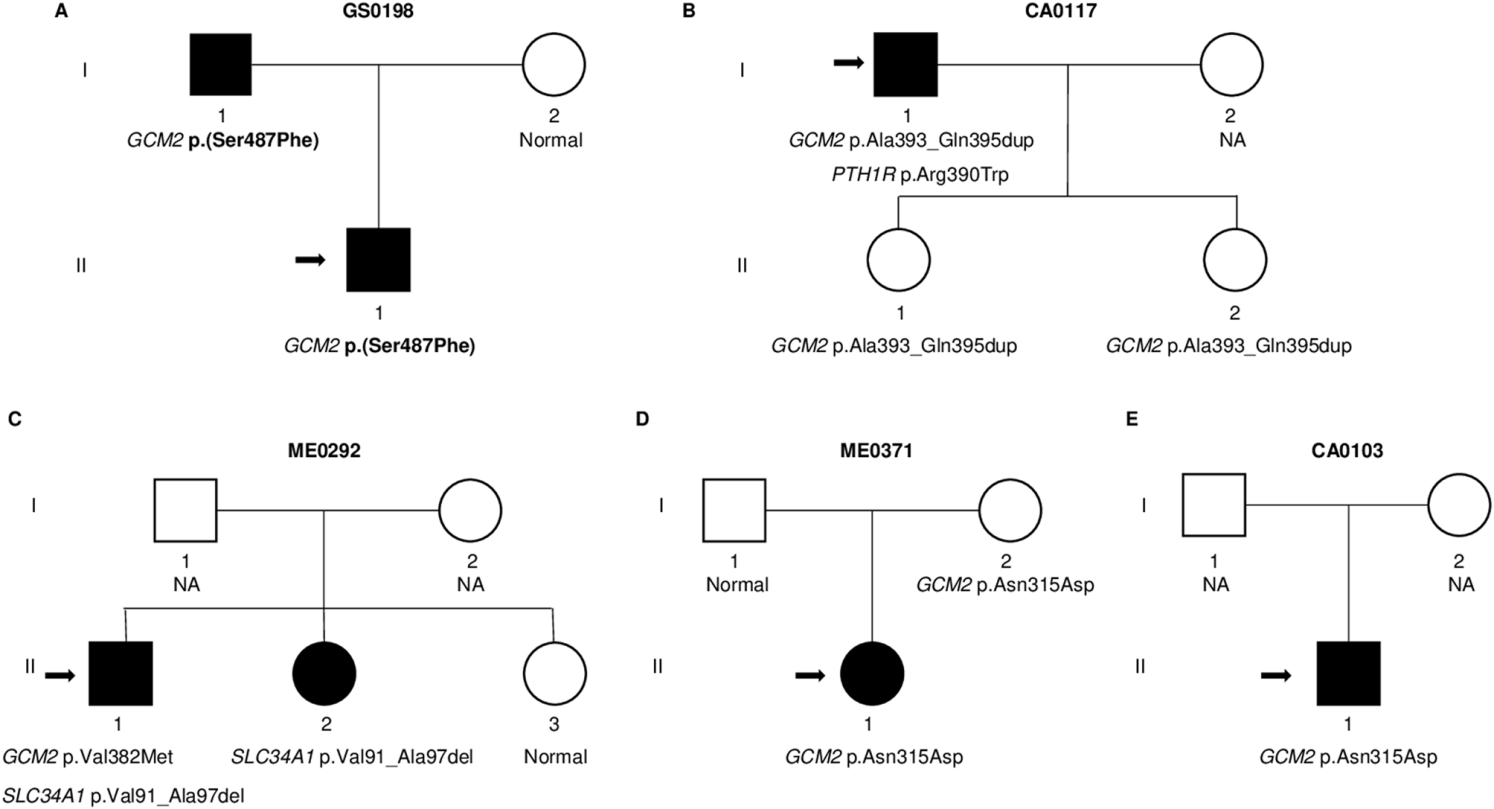
Pedigree of 5 families with variants in the *GCM2* gene. The novel variant is marked in bold. Index case are indicated by the arrows. Squares denote male family members, circles female family members, and solid symbols symptomatic subjects.

From the whole cohort, five index cases with a suspected disorder of calcium metabolism (GS0198, CA0117, ME0292, ME0371 and CA0103) presented with *GCM2* gene variants (Fig. 1).

Index case GS0198 was a 7-month-old child who was referred for evaluation of irritability and refusal to eat. He had suffered three episodes of seizures accompanied by rapid and involuntary muscle contractions. Laboratory results showed low serum Ca^2+^ (less than 5 mg/dL, reference range 8.5–10.4) and 25-hydroxyvitamin D (7.49 ng/mL, reference range 9–47), and high serum phosphate (11.7 mg/dL, reference range 2.6–4.8), whereas serum intact PTH levels (54 pg/mL, reference range 10–65) were within the normal range. In addition, he presented with moderate hypomagnesemia (1.39 mg/dL, reference range 1.7–2.5). While the mother had normal calcium and phosphate metabolism, proband's father had small stature (1.57 cm) and body segment disparity (short lower limbs). He had normal levels of serum Ca^2+^ (9 mg/dL), phosphate (3.18 mg/dL), magnesium (1.94 mg/dL), intact PTH (34.38 pg/mL) and 25-hydroxyvitamin D (30.4 ng/mL) (Fig. 1a).

Index case CA0117 was a 65-year-old male who had hypocalcemia and hypoparathyroidism. He had osteoarthritis and suffered tingling of fingers and toes. Moreover, he was diagnosed with glaucoma. Laboratory results showed low serum Ca^2+^ (5.4 mg/dL) and serum intact PTH (5.8 pg/mL), whereas 25-hydroxyvitamin D (24 ng/mL) and serum phosphate (4.3 mg/dL) were within the normal range. He was treated with calcium and vitamin D supplements. Regarding family history, there was no history of hypoparathyroidism or hypocalcemia (Fig. 1b).

Index case ME0292 was a 67-year-old male presenting with elevated serum intact PTH levels, hypophosphatemia, normocalcemia with vitamin D deficiency, hypercalciuria, and nephrolithiasis. Laboratory evaluation showed normal serum calcium (10.2 mg/dL), high intact PTH (95.6 pg/mL), low serum phosphate (2 mg/dL), and 25-hydroxyvitamin D levels of 11 ng/mL. In addition, he exhibited high urinary calcium excretion (386 mg/24 h, reference range in adult male < 300 mg/24 h). Furthermore, index case ME0292 had a personal history of ankylosing spondylitis, prostate gland enlargement, hepatic steatosis, and hyperlipidemia. He was diagnosed of primary hyperparathyroidism and parathyroid glands surgery was performed. Parathyroid hyperplasia of two superior glands was verified histologically and both were removed. However, after the surgical intervention, he continued with high intact PTH levels (113 pg/mL). He had a 61 year-old sister who had hyperparathyroidism (intact PTH 103 pg/mL) and nephrolithiasis as well (Fig. 1c). She had normal serum Ca^2+^ (9.4 mg/dL), normal serum phosphate (3.1 mg/dL) and exhibited slightly high urinary Ca^2+^ excretion (288 mg/24 h, reference range in adult women < 250 mg/24 h).

Index case ME0371 was a 17 year-old female who showed persistent hypercalcemia (serum Ca^2+^ 11.5 mg/dL), very high intact PTH (317 pg/mL), and normal serum phosphate (3.5 mg/dL). The patient exhibited high urinary Ca^2+^ excretion (329 mg/24 h) and slightly low urinary phosphate excretion of 329 mg/24 h (reference range 350–1000). The parathyroid scintigraphy with Technetium 99m-sestamibi (Tc99m-MIBI) was normal. Regarding family history, there was no history of hyperparathyroidism or hypercalcemia (Fig. 1d).

Finally, index case CA0103 was a 69 year-old male who showed normal serum Ca^2+^ (9.5 mg/dL), high intact PTH (140–200 pg/mL), and normal serum phosphate (3.2 mg/dL). The parathyroid scintigraphy with Technetium 99 m-sestamibi suggested a left parathyroid adenoma. The patient exhibited low urinary Ca^2+^ excretion (70 mg/24 h). Additionally, he had stage 3 chronic kidney disease which was stable in the last 15 years, and experienced an isolated episode of urinary lithiasis at 20 years of age. The last renal ultrasound performed was normal. Regarding family history, there was no history of hyperparathyroidism or chronic kidney disease (Fig. 1e).

### DNA analysis

We used the MagPurix instrument for genomic DNA extraction from peripheral blood leukocytes (Zinexts Life Science Corp., New Taipei City, Taiwan, R.O.C.). DNA purity and concentration were then determined using Qubit 2.0 fluorometer (Thermo Fisher Scientific, Waltham, Massachusetts, USA).

For Next-Generation Sequencing, a targeted panel for disorders of calcium and phosphorus metabolism was designed to include 65 genes associated with these disorders (*GNAS, STX16, PRKAR1A, PDE4D, PTHR1, PDE3A, PTHLH, CREB1, HDAC4, TRPS1, EXT1, HOXD13, ACVR1, GALNT3, LEP, LEPR, POMC, MC4R, TSHR, CASR, GNA11, AP2S1, MEN1, CDKN1A, CDKN1B, CDKN2B, CDKN2C, RET, CDC73, GCM2, CCND1, AIP, CTNNB1, EZH2, ZFX, DICER1, SDHB, SDHC, SDHD, CYP27B1, VDR, CYP2R1, CYP24A1, PTH, TBX1, NEBL, AIRE, GATA3, HADHB, TBCE, FAM111A, SOX3, DMP1, FGF23, SLC34A3, PHEX, SLC34A1, SLC9A3R1, ENPP1, TRPM6, CLDN16, CLDN19, ALPL, FXYD2* and *CNNM2*). The panel design was performed by Ion AmpliSeq Designer (Thermo Fisher Scientific). PubMed (https://www.ncbi.nlm.nih.gov/pubmed/) was consulted to select the genes included in the panel. Library preparation was done using the Ion Ampliseq Library Kit v2.0 (Thermo Fisher Scientific) according to manufacturer’s instructions. Samples were then sequenced using the Ion GeneStudio S5 System (Thermo Fisher Scientific). Base calling, read filtering, alignment to the reference human genome GRCh37/hg19, and variant calling were done using Ion Torrent Suite and Ion Reporter Software (Thermo Fisher Scientific).

Variants described in this article were tested by polymerase chain reaction (PCR), sequenced with fluorescent dideoxynucleotides (BigDye Terminator v3.1 Cycle Sequencing Kit, Life Technologies, Grand Island, NY, USA), and loaded onto an ABI3130xl Genetic Analyzer (Thermo Fisher Scientific).

DNA variants were named according to the Human Genome Variation Society guidelines (http://www.hgvs.org) and classified according to ACMG-AMP (American College of Medical Genetics and Genomics and the Association for Molecular Pathology) guidelines^10^.

As a measure of association between genotype and phenotype, we used Odds Ratio (OR). OR values above 1 mean there is an association between the variant and the risk of disease, while values below 1 mean there is a negative association between the variant and the risk of disease. If the 95% confidence interval for an OR includes 1, it means the results are not statistically significant^10^. We used the DJR Hutchon calculator for confidence intervals of odds ratio (http://www.hutchon.net/ConfidOR.htm).

## Results

**Figure 2:**
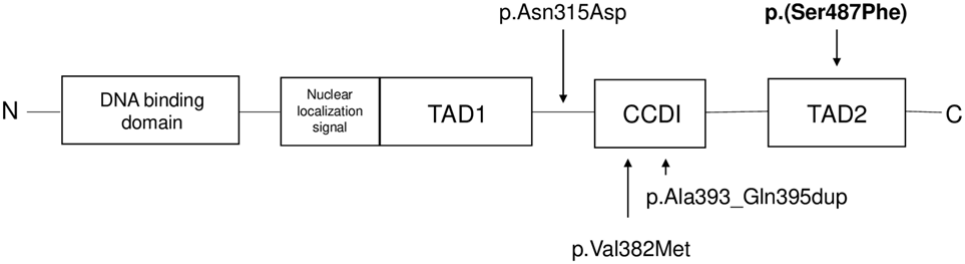
Schematic representation of the *GCM2* gene, representing the position of 3 missense variants and one short duplication. Variant marked in bold has not been reported to date. *TAD1* transcriptional activation domain 1, *TAD2* transcriptional activation domain 2, *CCID* C-terminal conserved inhibitory domain.

Regarding molecular diagnosis, we observed four variants in the *GCM2* gene. One novel variant of uncertain significance (c.1460C > T; p.(Ser487Phe)), and three previously reported variants; one variant of uncertain significance (c.943A>G; p.Asn315Asp), one likely pathogenic variant (c.1144G>A; p.Val382Met) and, finally, one benign variant (c.1185_1186insGCCTACCAG; p.Ala393_Gln395dup), all in the heterozygous state (Table 1 and Fig. 2).

**Table 1:** *GCM2* variants identified in patients.

Family	Number of individuals with the variant	Nucleotide change*	Amino acid change*	Exon	Domain	Phenotype	Variant class	References
ME0371	2	c.943A>G	p.Asn315Asp	5	Between TAD1 and CCID	Primary hyperparathyroidism	Uncertain significance	^6^
CA0103	1	c.943A>G	p.Asn315Asp	5	Between TAD1 and CCID	Primary hyperparathyroidism	Uncertain significance	^6^
ME0292	1	c.1144G>A	p.Val382Met	5	CCID	Primary hyperparathyroidism	Likely pathogenic	^11^
CA0117	3	c.1185_1186insGCCTACCAG	p.Ala393_Gln395dup	5	CCID	Hypoparathyroidsm	Benign	^12^
GS0198	2	c.1460C>T	p.(Ser487Phe)	5	TAD2	Hypocalcemia	Uncertain significance	This study

*Numbering is according to DNA sequence (Ensembl: ENST00000379491.5), all in heterozygous. *TAD1* transcriptional activation domain 1, *TAD2* transcriptional activation domain 2, *CCID* C-terminal conserved inhibitory domain.

Index case GS0198 had the p.(Ser487Phe) variant (Fig. 1a II1). According to ACMG-AMP guidelines, this variant is classified of uncertain significance (Table 2). This variant has not been found in the population database frequencies checked (GnomAD, ExAc, dbSNP, and 1000G), and occurs at a position within an important domain for the transcriptional function of the protein, near the C-terminus. Moreover, the serine residue at codon 487, located within the transcriptional activation domain 2 (TAD2), involves substitution of a polar neutral serine for the hydrophobic aromatic phenylalanine, probably disturbing the normal function of the protein. His father, who had small stature (1.57 cm) and short lower limbs, had the variant p.(Ser487Phe) in the heterozygous state (Fig. 1a I1).

**Table 2:** *GCM2* variants classification according to the ACMG guidelines.

Gene	Nucleotide change*	Amino acid change*	Selected criteria for pathogenic variants	Selected criteria for benign variants	Variant class
*GCM2*	c.943A>G	p.Asn315Asp	PS3: Well-established i*n vitro* functional studies supportive of a damaging effect on the gene product (20% more activity than wild-type protein)	BP6: Reputable source recently reports variant as benign but the evidence is not available to the laboratory to perform an independent evaluation (Reported in ClinVar as benign)	Uncertain significance
*GCM2*	c.1144G>A	p.Val382Met	PS3: Well-established in vitro functional studies supportive of a damaging effect on the gene product (2.1 times higher activity than wild-type protein) PM1: Located in a well-established functional domain (CCID) PP5: Reported as pathogenic	–	Likely pathogenic
*GCM2*	c.1185_1186insGCCTACCAG	p.Ala393_Gln395dup	PM1: Located in a well-established functional domain (CCID) PM4: Protein length changes as a result of in-frame duplication in a non-repeat region	BS1: Allele frequency is greater than expected for disorder BS3: Well-established in vitro functional studies show no damaging effect on protein function BP6: Reputable source recently reports variant as benign but the evidence is not available to the laboratory to perform an independent evaluation (Reported in ClinVar as benign and likely benign)	Benign
*GCM2*	c.1460C>T	p.(Ser487Phe)	PM1: Located in a well-established functional domain (TAD2) PM2: Absent from controls in Exome Sequencing Project, 1000 Genomes Project, or Exome Aggregation Consortium (ExAC)	–	Uncertain significance

*Numbering is according to DNA sequence (Ensembl: ENST00000379491.5 for the *GCM2* gene), all in heterozygous. *TAD2* transcriptional activation domain 2, *CCID* C-terminal conserved inhibitory domain, *PS* strong evidence of pathogenicity, *PM* moderate evidence of pathogenicity, *PP* supporting evidence of pathogenicity, *BS* strong evidence of benign impact, *BP* supporting evidence of benign impact.

We found two variants within the C-terminal conserved inhibitory domain CCID (p.Ala393_Gln395dup and p.Val382Met). Index case CA0117 had the variant p.Ala393_Gln395dup in the heterozygous state (Fig. 1b I1). This duplication, which is not in a repeat region, is located within an important regulatory region (the conserved inhibitory domain). It has been found in the population database frequencies checked with a low frequency (highest population Minor Allele Frequency (MAF): 0.01). Moreover, it has been observed in four adults in the homozygous state (GnomAD exomes). Furthermore, functional studies showed that this duplication has a transcriptional activity similar to wild-type protein^12^. In addition, we found this duplication in the patient’s two asymptomatic daughters in the heterozygous state as well (Fig. 1b II1, II2). This duplication is classified as benign according to ACMG-AMP guidelines (Table 2). Importantly, NGS analysis showed a second rare variant in index case CA0117. A heterozygous *PTH1R* variant (c.1168C>T; p.(Arg390Trp)) in exon 13 (Ensembl: ENST00000449590.6) was identified. This variant is not inherited by his two asymptomatic daughters. The *PTH1R* gene (MIM *168468) encodes the parathyroid hormone/parathyroid hormone related peptide receptor (PTH1R) that is a G-protein couple receptor for PTH and PTHLH (parathyroid hormone-like hormone) ^13^. This variant has a MAF < 0.01. The software Varsome ^14^ classified this variant as of uncertain significance according to ACMG-AMP guidelines (Table 3). PTH1R has 7 potential membrane-spanning domains and this variant occurs at a not conserved position located within a cytoplasmic topological domain (amino acids from 383 to 409) within this region (UniProtKB: Q03431) ^15^. Mutations in the *PTH1R* gene are known to cause Jansen's metaphyseal chondrodysplasia (MIM #156400), chondrodysplasia Blomstrand type (MIM #215045), Eiken syndrome (MIM #600002), and failure of tooth eruption (MIM #125350). However, our patient does not have clinical characteristics compatible with these diseases.

**Table 3:** Classification of *PTH1R* and *SLC34A1* variants according to the ACMG guidelines.

Gene	Nucleotide change*	Amino acid change*	Selected criteria for pathogenic variants	Selected criteria for benign variants	Variant class
*PTH1R*	c.1168C>T	p.Arg390Trp	PM2: Low frequency in ExAC (2/120788) and GnomAD-Exomes (5/251282) PP2: Missense variant in a gene that has a low rate of benign missense variation and in which missense variants are a common mechanism of disease	–	Uncertain significance
*SLC34A1*	c.272_292delTCCCCAAGCTGCGCCAGGCTG	p.Val91_Ala97del	PS3: Well-established in vitro functional studies supportive of a damaging effect on the gene product (functional analyses confirmed the impaired trafficking in HEK293 cells) PM4: Protein length changes as a result of in-frame deletions in a non-repeat region PP5: Reported as pathogenic	BP6: Reputable source recently reports variant as benign but the evidence is not available to the laboratory to perform an independent evaluation (ClinVar classifies this variant as Benign, rated 1 star)	Likely pathogenic

* Numbering is according to DNA sequence (Ensembl: ENST00000449590.6 for the *PTH1R* gene and Ensembl: ENST00000324417.5 for the *SLC34A1* gene), all in heterozygous; PS, Strong evidence of pathogenicity; PM, Moderate evidence of pathogenicity; PP, Supporting evidence of pathogenicity; BP, Supporting evidence of benign impact.

Index case ME0292 had the missense p.Val382Met variant located in the CCID (Fig. 1c II1). This variant has a MAF < 0.01 and was previously found in a parathyroid adenoma ^11^. Functional studies showed that this variant has 2.1 times higher transcriptional activity than wild-type ^6^. Therefore, p.Val382Met is considered an activation variant. In addition, NGS analysis showed a second rare variant in index case ME0292 in the *SLC34A1* gene (c.272_292del21; p.Val91_Ala97del) in the heterozygous state. The *SLC34A1* gene encodes the Sodium-dependent phosphate transport protein 2a (NaPi-2a, MIM * 182309) that is located in the apical membrane of renal proximal tubular cells ^16^. Mutations in this gene are associated with different clinical disease phenotypes; autosomal recessive form of infantile hypercalcemia type 2 (MIM # 616963) or Fanconi renotubular syndrome type 2 (MIM # 613388), and with autosomal dominant hypophosphatemic nephrolithiasis/osteoporosis type 1 (MIM # 612286). This small deletion in exon 4 (Ensembl: ENST00000324417.6) has been previously described in patients who presented with nephrolithiasis ^17–19^. Functional studies showed a reduction in the expression of this deletion in HEK293 cells, and a significantly reduction in phosphate transport compared with wild-type NaPi-2a in Xenopus oocytes ^19^. On the other hand, it is a relatively common deletion with a highest population MAF of 0.05. The proband's sister only had the p.Val91_Ala97del variant in *SLC34A1* in the heterozygous state. She presented with nephrolithiasis (Fig. 1c II2).

Finally, we found the p.Asn315Asp variant in the *GCM2* gene in the index cases ME0371 and CA0103 (Fig. 1d II1 and Fig. 1e II1, respectively) near the CCID. This variant has a MAF of 0.02 and it has been previously classified as benign or likely benign (ClinVar: VCV000712319.3). Functional studies showed that this variant has a 20% more transcriptional activity than the wild-type ^6^. Furthermore, it has been found in patients with parathyroid adenomas and hyperplasia ^20^. The asymptomatic mother of index case ME0371 had the p.Asn315Asp variant (Fig. 1d I2). In our cohort, we found two alleles with the variant p.Asn315Asp out of 88 alleles (44 patients with primary hyperparathyroidism analyzed by NGS). Compared with data of gnomAD for Non-Finnish European (481/129204), we observed an enrichment of this variant in patients with primary hyperparathyroidism in our cohort [Odds Ratio (OR): 6.1049, Confidence Interval IC95% (1.49–24.86)]. According to ACMG-AMP guidelines, we classified this variant as of uncertain significance (Table 2).

## Discussion

In this study, we describe five families who had variants in the *GCM2* gene. The complete genetic study revealed one novel variant of uncertain significance (c.1460C>T; p.(Ser487Phe)), and three previously reported variants; one of uncertain significance (c.943A>G; p.Asn315Asp), one likely pathogenic variant (c.1144G>A; p.Val382Met) and, finally, one benign variant (c.1185_1186insGCCTACCAG; p.Ala393_Gln395dup), all in the heterozygous state. In addition, the genetic study revealed other two variants located in the *PTH1R* and *SLC34A1* genes, of uncertain significance and likely pathogenic respectively, both in the heterozygous state as well (Table 3).

So far, according to the Human Gene Mutation Database (http://www.hgmd.cf.ac.uk), 14 variants in the *GCM2* gene have been reported associated with hypoparathyroidism. These 14 variants were considered as loss-of-function mutations. In our genetic study, two variants in the *GCM2* gene were found in two patients with hypocalcemia (GS0198) and hypoparathyroidism (CA0117). Index case GS0198 and his father had the missense p.(Ser487Phe) variant in the heterozygous state located within the TAD2 (Fig. 2). As far as we know, only another missense mutation within the TAD2 (p.Asn502His) has been reported ^21^. Furthermore, a dominant-negative effect produced by two small deletions affecting the TAD2, p.(His465Thrfs*66) and p.(Pro467Glnfs*64), has been described by other studies ^22^. The p.Asn502His variant showed a reduction in transactivation and it was found in the heterozygous state in one patient diagnosed at 5 days of age, presenting with hypocalcemia, hyperphosphatemia, hypomagnesemia, low 25-OH vitamin D levels and normal serum intact PTH levels. The same clinical features were observed in our index case GS0198 who had the p.(Ser487Phe) variant. Moreover, the p.Asn502His variant showed a dominant-negative effect ^21^. In the family previously described, the proband’s father had the p.Asn502His variant in the heterozygous state as well ^21^. He only presented with finger paresthesia and mild hypocalcemia (8.14 mg/dL), while index case GS0198’s father had small stature and body segment disparity. These two variants, p.(Ser487Phe) and p.Asn502His, could produce a similar effect in the protein in the heterozygous state.

Index case CA0117 had the p.Ala393_Gln395dup variant, which is located within the CCID (Fig. 2). As far as we know, only one polymorphism showing a reduced transcriptional activity (10% reduction) has been described in the heterozygous state in this domain (p.Lys388Gln) ^12^. The p.Ala393_Gln395dup duplication observed in index case CA0117 and his two asymptomatic daughters produces an extension of the inhibitory region. Functional studies showed a similar activity than the wild-type protein ^12^. Moreover, this duplication is maybe common to be a pathogenic mutation (1% in some populations). On the other hand, this duplication is enriched in our cohort [OR: 59.15 (IC95% 13.92–251.2)] compared with a control population of Non-Finnish European [gnomAD (168/129188)]. We performed genetic analysis by NGS in 127 patients (254 alleles). Thirteen had hypocalcemia/hypoparathyroidism (26 alleles). Only two patients with hypocalcemia (one of them is not included in the manuscript) had the p.Ala393_Gln395dup duplication in the *GCM2* gene (2/26).

Three gain-of-function mutations located in the CCID (p.Leu379Gln, p.Lys388Glu and p.Tyr394Ser) with 3.3, 2.1 and 2.4 times higher activity in the heterozygous state, respectively ^12^ and two disease-associated polymorphisms (p.Arg59Cys and p.Tyr282Asp) have been previously described associated with hyperparathyroidism. In our cohort, we found one likely gain-of-function mutation (p.Val382Met). The p.Val382Met variant, which is located in the CCID, was previously reported in a parathyroid adenoma ^11^. Functional studies performed in the CCID demonstrated that this variant has 2.1 times higher activity than wild-type ^6^. Therefore, the p.Val382Met variant caused the parathyroid hyperplasia observed in index case ME0292.

A few variants out of the CCID, p.Gln330Leu and p.Arg406Gln, have been reported in patients with primary hyperparathyroidism ^20^. Functional analysis in p.Asn315Asp, present in our cohort, and also located out of the CCID (Fig. 2), showed that it has a 20% more transcriptional activity than the wild-type ^6^. Furthermore, it has been found in patients with parathyroid adenomas and hyperplasia ^20^. Thus, we hypothesize that the p.Asn315Asp variant may cause the hyperparathyroidism present in index cases ME0371 and CA0103. On the other hand, the asymptomatic mother of index case ME0371 had the p.Asn315Asp variant, and this is in line with the phenotype variability within the family previously observed. Instead, penetrance seems to be low, so it has been suggested that the majority of individuals with gain-of-function variants in the *GCM2* gene will not develop a parathyroid adenoma ^23^.

Importantly, the genetic study showed a second variant in index case CA0117. The p.(Arg390Trp) variant, located in the heterozygous state in the *PTH1R* gene, is not inherited by his two daughters. This variant is located in a cytoplasmic region between the 5 and 6 transmembrane domains. Pathogenic mutations within the transmembrane domain have been associated with Murk Jansen Chondrodysplasia. Thus, patients with recessive mutations presented with mild hypercalcemia, hypophosphatemia, low intact PTH levels, hypercalciuria, bone dysplasia, kidney stones, bowing and osteopenia. On the other hand, patients with dominant mutations presented with a milder form of the disease, with less severe skeletal and mineral ion abnormalities ^24^. Moreover, it has been described that some polymorphisms in the *PTH1R* gene can determine the sensitivity of the kidney and bone to the catabolic or anabolic action of PTH ^25^. This variant is not located in an important domain for binding to PTH, PTHLH or the signalling initiator G protein and the patient does not present symptoms compatible with diseases associated with pathogenic variants in the *PTH1R* gene. Therefore, it is unlikely to influence the phenotype of the patient.

On the other hand, index case ME0292’s sister presented with high intact PTH levels and nephrolithiasis without other remarkable symptoms. She does not have the p.Val382Met variant in the *GCM2* gene. However, we observed another variant in the *SLC34A1* gene in the index case ME0292 and his sister in the heterozygous state (p.Val91_Ala97del). Despite the p.Val91_Ala97del deletion is frequent in general population (MAF of 0.01), functional studies demonstrated that it exhibits a significantly reduced phosphate transport compared with wild-type, and considering the high prevalence of people globally being affected by kidney stones (1–15%)^26^, we cannot exclude that this deletion may cause nephrolithiasis in family ME0292.

Our study shows the utility of NGS in unravelling the genetic origin of disorders in the calcium and phosphorus metabolism. Moreover, our results confirmed GCMb as an important genetic element for the maintenance of calcium homeostasis, as it interacts with genes involved in the calcium metabolism such as *CASR*, modifying its expression, and *GATA3* and *MAFB* modifying PTH expression ^4^. However, the penetrance seems to be low probably because some mechanisms of compensation occur ^20^.

In conclusion, this study identified four variants in the *GCM2* gene, of which one was novel (p.(Ser487Phe)) and classified as a variant of uncertain significance. Further studies aimed at the functional characterization of this variant will be of help in defining the hypothesized pathogenic role.
